# Workload and emerging challenges of community health workers in low- and middle-income countries: A mixed-methods systematic review

**DOI:** 10.1371/journal.pone.0282717

**Published:** 2023-03-13

**Authors:** Tigist Astale, Tsegereda Abebe, Getnet Mitike

**Affiliations:** International Institute for Primary Health Care-Ethiopia, Addis Ababa, Ethiopia; Spreeha Foundation Bangladesh / The University of Sydney / The University of Southern Queensland, AUSTRALIA

## Abstract

**Background:**

Community health workers (CHWs) play an important role in improving access to health services to a broader population; particularly to communities living in remote areas. However, the productivity of CHWs is affected by the workload they have. We aimed to summarize and present CHWs’ perceived workload in low-and middle-income countries (LMICs).

**Methods:**

We searched three electronic databases (PubMed, Scopus, and Embase). A search strategy customized for the three electronic databases was developed using the two key terms of the review (CHWs and workload). Primary studies conducted in LMICs that explicitly measured workload of CHWs and published in English were included, without date restrictions. Methodological quality of the articles was assessed by two reviewers independently using mixed-methods appraisal tool. We applied a convergent integrated approach to synthesize the data. This study is registered on PROSPERO, number CRD42021291133.

**Results:**

Of 632 unique records, 44 met our inclusion criteria, and 43 (20 qualitative, 13 mixed-methods, and 10 quantitative studies) passed the methodological quality assessment and were included in this review. In 97.7% (n = 42) of the articles, CHWs reported that they have a high workload. Having multiple tasks was the most commonly reported subcomponent of workload, followed by lack of transport; which was reported in 77.6% (n = 33) and 25.6% (n = 11) of the articles respectively.

**Conclusion:**

CHWs in LMICs reported that they have a high workload; mainly related to having to manage multiple tasks and the lack of transport to access households. Program managers need to make careful consideration when additional tasks are shifted to CHWs and the practicability to be performed in the environment they work in. Further research is also required to make a comprehensive measure of the workload of CHWs in LMICs.

## Introduction

In many low- and middle- income countries (LMICs), primary health care approach is adopted through the deployment of community health workers (CHWs) [1, 2]. Since the Alma-Ata Declaration, a well-designed CHWs approach within the primary health care system has been considered to be an effective strategy for achieving universal health coverage (UHC). Systematic reviews in different settings also reported the effectiveness of CHWs in providing a range of promotive, preventive and basic curative health services [3–7].

As task-shifting strategies become widely implemented in many countries, CHWs are assigned for additional tasks through in-service trainings. As a result, the health services provided by CHWs are evolving through time with a wider scope and complexity [8, 9], which could, in turn, reduce the level of productivity [10–13], and increase the risks of turn over intention [14]. Higher workload among rural health workers has also been associated with increased level of burnout [15].

Community health workers’ productivity is largely determined by the level of manageable workload in terms of tasks [9]. However, despite the growing recognition of CHWs’ critical role in increasing access to health services for underserved communities, little attention has been given to how much work responsibility can CHWs take before a work overload negatively affects their productivity. A recent review by WHO on CHW programs highlights that the tasks assigned to CHWs need to align with the level of training and appropriate remuneration and that attention should be given to the views of CHWs in terms of their work responsibilities [16, 17].

Community health worker workload is a multifactorial concept that can be described by the interplay of the number and organization of tasks and the catchment area (number of households and their geographic distribution [9]). This systematic review will present the views of CHWs on their perceived workload, in terms of the specific subcomponent of workload that is mostly contributing for their workload. The findings could inform program managers to design task-shifting strategies that ensure realistic workload and contribute for productive CHWs and sustainable CHW programs.

The definition of CHWs varies across countries and programs. For the purpose of this review CHWs are defined as health workers based in communities (i.e. conducting outreach from their homes and beyond primary health care facilities or based at peripheral health posts that are not staffed by doctors or nurses), who are either paid or volunteer, who are not professionals, and who have fewer than 2 years training but at least some training, if only for a few hours [17].

## Methods

### Data sources and search strategy

This systematic review was conducted following Preferred Reporting Items for Systematic Reviews and Meta-Analyses (PRISMA) guidelines. Systematic literature search was performed on 3 electronic databases (PubMed, Embase, and Scopus) by two individuals. No limits were applied on dates of publication. A broad search strategy was developed that incorporates the two main components of the review (community health workers and workload). Similar possible terminologies for these two components were considered in developing the search strategy. The search terms used for systematic search in each database is presented below.

**PubMed**: ("Workload"[Mesh] OR workload*[Title/Abstract] OR burden*[Title/Abstract] OR "time allocation*"[Title/Abstract] OR "training need*"[Title/Abstract] OR "training requirement*"[Title/Abstract]) AND ("community health workers"[MeSH Terms] OR CHW[tiab] OR CHWs[tiab] OR "Community health worker*"[Title/Abstract] OR "community health volunteer*"[Title/Abstract] OR "health volunteer*"[Title/Abstract] OR "health promoter*"[Title/Abstract] OR "village health worker*"[Title/Abstract] OR "primary health worker*"[Title/Abstract] OR "rural health worker*"[Title/Abstract] OR "community health officer*"[Title/Abstract] OR "women development army"[Title/Abstract] OR "development army"[Title/Abstract] OR "women development armies"[Title/Abstract] OR "development armies"[Title/Abstract] OR "health extension worker*"[Title/Abstract] OR "voluntary health worker*"[Title/Abstract] OR "volunteer health worker*"[Title/Abstract] OR "lay health worker*" [Title/Abstract] OR "community health assistant*"[Title/Abstract] OR "community health aide"[Title/Abstract] OR "community health aides"[Title/Abstract] OR "community volunteer*"[Title/Abstract] OR "village health volunteer*" [Title/Abstract]) OR accredited social health activist*[tiab] OR ASHA worker*[tiab] OR auxiliary health worker*[tiab] OR health auxiliar*[tiab] OR barefoot doctor*[tiab] OR community health practitioner*[tiab] OR medical auxiliar*[tiab].

**Embase**: ’workload’/exp OR ‘workload’:ti,ab,kw OR ‘work load’:ti,ab,kw OR burden*:ti,ab,kw OR ’time allocation*’:ti,ab,kw OR ’training need*’:ti,ab,kw OR ’training requirement*’:ti,ab,kw AND ’health auxiliary’/exp OR ’community health volunteer*’:ti,ab,kw OR ’health volunteer*’:ti,ab,kw OR ’health promoter*’:ti,ab,kw OR ’village health worker*’:ti,ab,kw OR ’primary health worker*’:ti,ab,kw OR ’rural health worker*’:ti,ab,kw OR ’community health officer*’:ti,ab,kw OR ’women development army’:ti,ab,kw OR ’development army’:ti,ab,kw OR ’women development armies’:ti,ab,kw OR ’development armies’:ti,ab,kw OR ’health extension worker*’:ti,ab,kw OR ’voluntary health worker*’:ti,ab,kw OR ’volunteer health worker*’:ti,ab,kw OR ’lay health worker*’:ti,ab,kw OR ’community health assistant*’:ti,ab,kw OR ’community health aide’:ti,ab,kw OR ’community health aides’:ti,ab,kw OR ’community volunteer*’:ti,ab,kw OR ’village health volunteer*’:ti,ab,kw OR ’accredited social health activist*’:ti,ab,kw OR ’ASHA worker*’:ti,ab,kw OR ’auxiliary health worker*’:ti,ab,kw OR ’health auxiliar*’:ti,ab,kw OR ’barefoot doctor*’:ti,ab,kw OR ’community health practitioner*’:ti,ab,kw OR ’medical auxiliar*’:ti,ab,kw.

**Scopus**: (workload* OR “work load*” OR burden* OR "time allocation*" OR "training need*" OR "training requirement*") AND ("Community health worker*" OR "community health volunteer*" OR CHW OR CHWs OR "health volunteer*" OR "health promoter*" OR "village health worker*" OR "primary health worker*" OR "rural health worker*" OR "community health officer*" OR "women development army" OR "development army" OR "women development armies" OR "development armies" OR "health extension worker*" OR "voluntary health worker*" OR "volunteer health worker*" OR "lay health worker*" OR "community health assistant*" OR "community health aide" OR "community health aides" OR "community volunteer*" OR "village health volunteer*" OR "accredited social health activist*" OR "ASHA worker*" OR "auxiliary health worker*" OR "health auxiliar*" OR "barefoot doctor*" OR "community health practitioner*" OR "medical auxiliar*").

We conducted initial search on Dec 21/2021 that resulted in 619 articles on PubMed. The search was updated on March 17, 2022 to include additional search terms on PubMed and including two additional databases (Scopus and Embase).

### Study selection and critical appraisal

**Fig 1:**
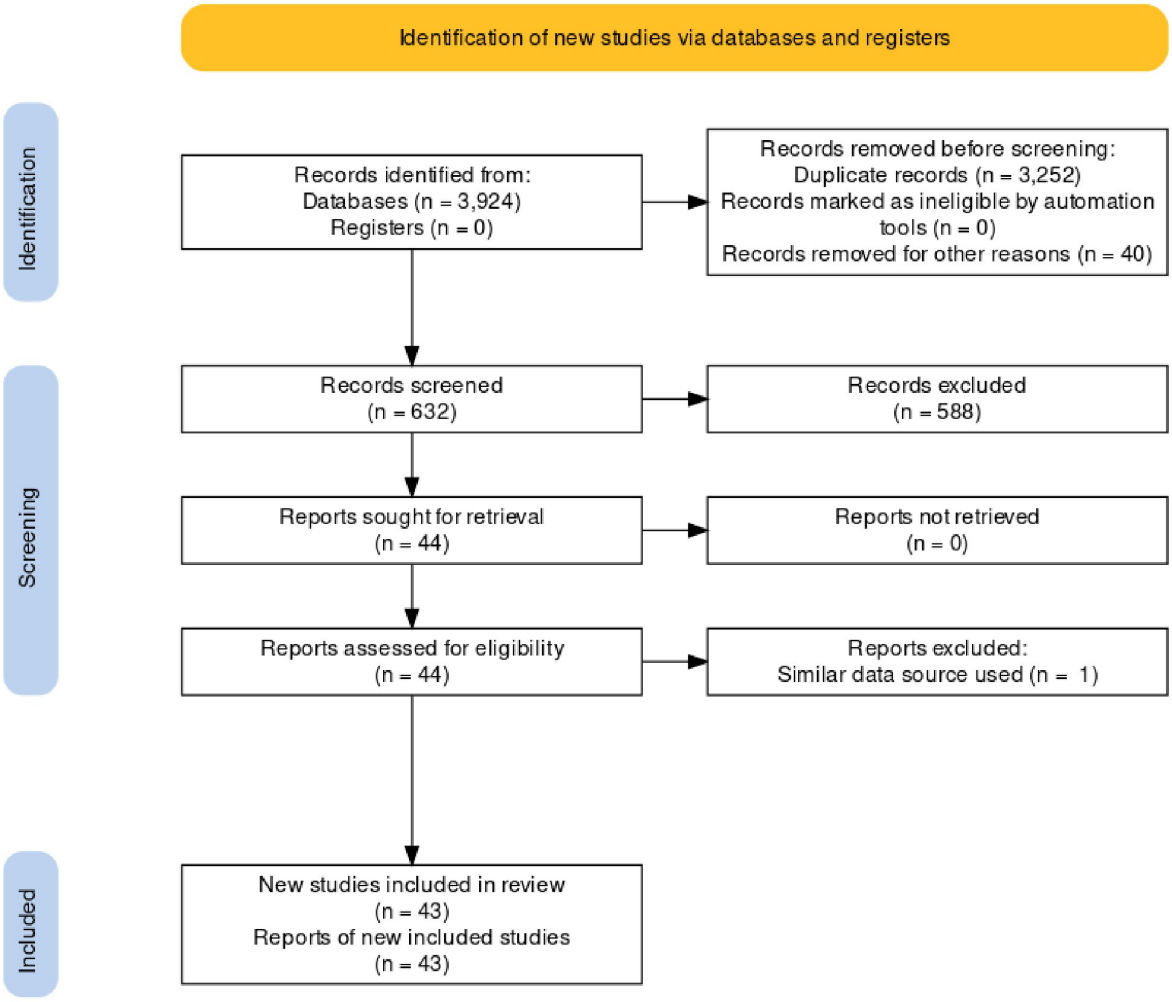
Flow diagram for database searches and study selection.

First, all study articles found using the search terms from the 3 electronic databases were imported to Endnote version 9 and duplicate articles were removed. Next, articles not in English language, study protocols, review articles, and overviews were removed. The titles and abstracts were then screened by two authors (TA and TA) independently based on the predefined eligibility criteria (Quantitative, Qualitative, and mixed-method study articles conducted in LMICs that explicitly reported on workload of CHWs). Finally, two authors (TA and TA) performed critical appraisal for the selected full articles using The Mixed Methods Appraisal Tool (MMAT) version 2018 [18]. Overall score from the ratings of each criterion was not calculated as it is not recommended when applying the MMAT tool [18]. Rather, the reviewers discussed on the assessments of each study based on each criterion. Consensus was reached on the disagreements of the ratings through discussion. Forty-three articles passed the quality assessment stage and were included into the final data extraction and analysis. The Study selection process is presented below using PRISMA flow diagram (Fig 1).

### Data extraction

Excel data extraction form was developed that captured the following contents: first author, publication year, country where the study was conducted, main objective, study design/type, type of community health worker (paid or volunteer), sample size, general perceived workload and subcomponents of workload. Data was extracted by one reviewer. Any measure of workload was eligible for inclusion. Results on workload may be reported either as a general perceived workload or in terms of different subcomponents of workload. The reported workload was extracted as it is reported in each study and categories were made by the reviewers.

### Data synthesis and integration

A convergent integrated approach was applied to synthesize quantitative and qualitative data concurrently [19]. To allow integration of quantitative data with qualitative data, quantitative data was transformed into qualitative data(“qualitized”) by extracting quantitative data and converting it into textual descriptions. Qualitative data were coded and categorized into themes. The qualitative and “qualitized” data were then compared to create integrated themes.

## Results

We identified 632 unique records; 44 met our inclusion criteria. Forty-three articles were of good methodological quality and included in to the final data extraction and analysis. One article [20] did not pass the methodological quality assessment because it used similar data source with one of the included studies. Detailed characteristics of the reviewed studies is provided as a S1 File. In summary, 20 studies were qualitative, 13 mixed-methods, and 10 quantitative studies. Almost half of the studies (n = 22) addressed volunteer CHWs, and the remaining articles (n = 21) addressed paid CHWs. The integrated findings and sample qualitative and “qualitized” data are summarized in S1 Table.

In almost all of the included studies (except one study), CHWs reported that they have a considerable amount of workload. The finding in this one article [21] differs from the other included articles in that additional tasks (on nutritional interventions) was considered as positive rather than its effect on increasing burden on them. The perceived workload reported from the other 42 articles was comparable among paid and volunteer CHWs, and in all quantitative, qualitative, and mixed-methods studies.

Further looking at the workload experiences reported in each study, four subcomponents (integrated findings) were identified: tasks; lack of transport; catchment area; and competing socio-cultural and economic demands. The integrated findings are explored below.

### Tasks

The number of tasks assigned to CHWs was the most frequently mentioned aspect that contributed for CHW’s perceived workload. In 77.6%(n = 33) of the included articles, CHWs reported that they feel overburdened by the number of tasks assigned to them. The workload related to tasks was assessed in the 33 articles differently. In the 29 articles [22–50], CHWs directly responded their views in terms of multiple tasks contributing for their workload where as in the 4 articles [51–54], CHWs reflected on their workload in terms of working for long hours (which could indirectly be linked to having multiple tasks). These findings were reported in a similar pattern in all quantitative, qualitative, and mixed-methods studies included in this review. Besides, there was no contracting finding among volunteers and paid CHWs in terms of this theme.

### Lack of transport

In 11 (25.6%) of the reviewed articles, lack of transport was reported as one of the aspects that contributed for their high perceived workload [10, 24, 25, 29, 33, 49, 50, 54–57]. This theme emerged mostly from mixed-methods(n = 5) and qualitative studies (n = 5), and only one quantitative study contributed to this theme. Lack of transport was a common finding in both volunteer and paid CHWs. Both types of CHWs reported that they walk for long distances because of the remoteness of the households and that lack of transport increased their workload.

### Catchment area/number of households

In 6(14%) of the reviewed articles, CHWs reported that they serve a high number of households under their catchment area which contributed to their increased workload [10, 56, 58–61]. High number of households was assessed differently by which serving more than 30 households and 240 households were considered equally as high.

### Competing economic and socio-cultural demands

In 6 articles (14%), CHWs reported that the work compromised their socio-cultural and economic demands in their daily life [29, 39, 40, 49, 56, 60]. This was particularly reported by volunteer CHWs instead of paid CHWs. The volunteer CHWs in these 4 articles [29, 40, 56, 60] indicated that the more they spend time on the community work, the more they face challenges in their households in terms of missing family caring responsibility and losing money-generating activities to support their family. This theme arose from CHWs in both quantitative and qualitative studies. In one qualitative study, CHWs indicated that they are doing time-consuming voluntary work that deserved monthly salary, and they were initially poor and now getting poorer after they joined due to out -of- pocket expenditure for work related activities [29]. This finding was complemented by quantitative data whereby 37% of volunteer workers have high number of households under their catchment area and this resulted in time-aways from their important economic endeavors [60].

## Discussion

In this mixed-methods systematic review, we observed that CHWs experienced a considerable amount of perceived workload. The perceived workload was comparable among paid and volunteer CHWs. Further looking at the subcomponents of workload, the major elements of workload were reflected in terms of multiple tasks, lack of transport, large catchment area, and competing socio-cultural and economic demands (mainly reported by volunteer CHWs).

A common finding in our review was around multiple tasks being the major aspect of workload. Community health workers in 77.6% of the reviewed articles reported that they are required to perform a very broad-range of tasks ranging from multiple health programs to agricultural and political sectors. While there is no known fair/ideal number of tasks that would be manageable by CHWs, too many roles could have a negative impact on level of productivity [9]. It was further noted in the reviewed articles that CHWs perform tasks that are not described in their job descriptions and sometimes perform tasks that they are not trained for or do not have the required knowledge and skill. Reports show that when CHWs are overwhelmed by a broad-range of tasks, they tend to select few that they prefer to do and ignore the others [62], which could in turn, affect the overall success of the CHW program. A recent review by WHO working group also emphasizes the importance of clearly defined tasks that align with the appropriate renumeration and support [17]. On the other hand, how well the tasks are organized or integrated is crucial. Even though the number of tasks is high, integrating the timing and approach of implementation may decrease workload and increase productivity [9].

Lack of transport was the other common subcomponent of workload which was reported by 25.6% of the reviewed articles. Most of the CHWs in these articles reported a feeling of exhaustion to perform their duties after walking for long hours to cover their target households. The design of community health programs in several countries has been mainly focused on the amount of time that would be required to finish a given task, and less attention has been given to the effect of time spent to access the target households on productivity. Although we found limited data on the link between transport availability and productivity, it appears that lack of attention to travel time and lack of transport to access the households is a key factor for the productivity of CHWs. Furthermore, a combined effect of lack of transport and number of households might have implications on productivity.

We found that perceived workload among paid and volunteer CHWs was comparable; both groups of CHWs reported that they have high workload. However, looking at the subcomponents of workload, competing socio-cultural and economic demands was an important element to volunteer CHWs than paid CHWs. Unlike the paid CHWs, unpaid volunteer CHWs cited that they are also expected to do income generating activities to support their families. As such, expecting volunteer CHWs to work for many hours per week creates extra burden, which would eventually lead them to leave their responsibility [63]. In some situations, volunteer CHWs have out of pocket expenditures for a work-related activity that led them to get poorer after joining the volunteer work [29].

We included articles from three widely used electronic databases, and considered both paid and volunteer CHWs. However, the findings from this review should be interpreted with the following limitations. First, we did not include articles that were not published in English, articles that were not published in peer-reviewed journals, and articles that we do not have full access. Second, perceived workload was assessed in the reviewed articles differently. Some of the articles were focused on the task, while the others focus on the hours worked per week, or whether lack of transport was a challenge in their day-to-day activities. As such, one subcomponent of workload was not mentioned in the article does not necessarily mean it was not a problem in those interviewed participants. Number of households under CHWs catchment area was also categorized differently in that the number of households in one setting may be categorized as high whereas it is categorized as low in another setting. A comprehensive investigation of workload using high-quality measurement instruments would be required to solve this gap.

In conclusion, this review highlighted that for many CHWs, their workload is overwhelming. In addition to the increasing number of tasks that the CHWs are expected to perform, lack of transport further intensified their workload. While acknowledging the benefit of decentralization and task shifting strategy to increase access to health services to remote areas, program managers need to make a careful consideration as to how the additional tasks can be well integrated into the existing responsibilities of CHWs. It is also critical that when workload of CHWs is investigated all the integral components of workload (number of tasks, catchment area/number of households, and availability of transport) need to be considered and not solely on the number of tasks per CHW.

## Supporting information

S1 Table Summary of integrated findings related to workload. (DOCX)

S1 File Detailed characteristics of the reviewed articles. (DOCX)

S2 File Completed PRISMA 2020 checklist. (DOCX)
