# Supplementary material for: Workload and emerging challenges of community health workers in low- and middle-income countries: A mixed-methods systematic review
Source: PLoS One. 2023 Mar 13;18(3):e0282717. doi: 10.1371/journal.pone.0282717 (PMC10010520; doi:10.1371/journal.pone.0282717)
Supplement: S1 Table — (DOCX) [file pone.0282717.s001.docx]

**Table 1. Summary of integrated findings related to workload**

| **Integrated finding** | **Contributing studies n** | **Qualitative data example** | **“Qualitized”data example** |
| --- | --- | --- | --- |
| Tasks | Qualitative studies=8  Quantitative studies=7  Mixed-methods studies=18 | “I feel overwhelmed, because in addition to the work of CHA, I do many other things. They give me all the bad tasks. They ask me to work outside my area” [24] | Majority (~88% of community health workers worked greater or equal to 40 hours per week) [53] |
| Lack of transport | Qualitative studies=5  Quantitative studies=1  Mixed-methods studies=5 | “There’s sometimes a long distance that we must walk, a long distance, and sometimes the clinic don’t have the medication” [54] | Majority (71.3%) of CHWs reported lack of transport as one of their constraints as a CHW. |
| Catchment area | Qualitative studies=1  Quantitative studies=2  Mixed-methods studies=3 | “First of all, the challenge is that, my village is very large consisting of five sub-villages. I walk by foot alone to provide services to the community” [61] | Volunteers who served many people experienced a higher level of Burden [59]. |
| Competing economic and socio-cultural demands | Qualitative studies=3  Quantitative studies=1  Mixed-methods studies=2 | “You can take someone to the hospital, so the business that you run will not open that day…when you come back the children want something to eat, you don't have money because you did not work that day” [49] | 37% of community health workers served more than the recommended number of households. Higher number of households a CHV oversees results into increased workload for these CHVs who are likely working on  part-time basis—“volunteering.”[60] |
