# Supplementary material for: Workload and emerging challenges of community health workers in low- and middle-income countries: A mixed-methods systematic review
Source: PLoS One. 2023 Mar 13;18(3):e0282717. doi: 10.1371/journal.pone.0282717 (PMC10010520; doi:10.1371/journal.pone.0282717)
Supplement: S1 File — (DOCX) [file pone.0282717.s002.docx]

| **SN** | **First author, Year of publication** | **Country** | **Main objective** | **Type of study/study design** | **Sample size** | **Type of CHW (paid/volunteer)** |
| --- | --- | --- | --- | --- | --- | --- |
| 1 | A. Kebriaei, 2009 | Iran | To investigate job satisfaction among health care workers | Quantitative | 76 | paid |
| 2 | Achamyelesh G, 2020 | Ethiopia | To explore community acceptance and utilization of MCH services provided by CHWs/HEWs, best practices, barriers, and challenges | Qualitative | 49(including CHWs, community member, CHW supervisors) | paid |
| 3 | Adelaide M., 2021 | Kenya | To examine socio economic challenges that affect the volunteer work of community health volunteers | Qualitative | 8 key informant interview, 10 focus group discussions that involve community health volunteers | volunteer |
| 4 | Anand K., 2021 | India | To assess and explore  ASHAs’ perspectives of their workload alongside that  of local healthcare colleagues in both rural and tribal  contexts. | Mixed methods | 67 | volunteer |
| 5 | Anthony K, 2018 | Kenya | To measure CHWs attrition and its predictors | Mixed methods | 363 for the quantitative; and 12 for qualitative study | volunteer |
| 6 | Armande K, 2016 | Burkinafaso, Nigeria, Uganda | To investigates factors influencing CHW motivation and retention in health service delivery | Mixed methods | 206(for quantitative); 48(for qualitative) | volunteer |
| 7 | Cuixia Ge, 2011 | China | To determine level of job satisfaction among CHWs | Quantitative | 2100 | paid |
| 8 | Denise M, 2018 | Brazil | To understand the perception of Community Health Agents regarding their workloads | Qualitative | 14 | paid |
| 9 | Geofrey K, 2015 | Uganda | To establish the roles of VHTs, the service gaps encountered and the measures in place to address these gaps | Mixed methods | 150 for quantitative; 14 for qualitative | volunteer |
| 10 | Gertrude N., 2017 | Uganda | To explore knowledge levels of CHWs, describes the coverage of home visits, and shares lessons learnt from setting up and implementing the CHW strategy. | Mixed methods | 10 key informant and 15 CHWs for qualitative | volunteer |
| 11 | Gladys K., 2020 | South Africa | To explore CHWs’ motivation to deliver systematic household contact tuberculosis (TB) investigation (SHCI). | Quantitative | 235 | paid |
| 12 | Hashem H., 2020 | Iran | To explain the comprehensive model of health education barriers of health‑care system | Qualitative | 21 other staff; 26 CHWs | paid |
| 13 | James B., 2014 | Uganda | To assess factors influencing performance of CHWs managing malaria, pneumonia and diarrhoea under the Integrated Community Case Management (iCCM) programme | Quantitative | 336 | volunteer |
| 14 | Janna M., 2020 | Rwanda | (1) calculate the opportunity costs incurred by CHWs, (2) characterize factors contributing positively and negatively to CHW motivation, job satisfaction and service delivery, and (3) describe CHW perspectives on strategies for improved satisfaction and service delivery. | Mixed methods | 145 | volunteer |
| 15 | Jeanine C., 2014 | Rwanda | To assess the capacity of CHWs and the factors affecting the efficiency and effectiveness of the CHW programme, as perceived by the CHWs and their beneficiaries. | Mixed methods | 108 CHWs; 36 beneficiaries | volunteer |
| 16 | JG J., 2010 | Haiti | To evaluate CHW role in the health system in the context of both HIV and non-HIV related services, as well as challenges and facilitating factors they faced in this role. | Qualitative | 462 | paid |
| 17 | Joan N., 2012 | Uganda | To compare the performance of CHWs in the dual management of malaria and pneumonia vs CHW management of malaria alone in children under five and to assess the factors influencing CHW performance | Mixed methods | 125 | volunteer |
| 18 | Joana R., 2015 | Democratic Republic of Congo, Ghana, Senegal, Uganda and Zimbabwe | To explore the current use of practices for attraction, retention and performance management of CHWs in five African countries | Qualitative | FGD with 13 CHWs; interview with 31 others who are related to the work of CHWs; 43 document review. | volunteer |
| 19 | Kenneth M., 2018 | Ethiopia | To examine deprivation and wellbeing in Ethiopia’s Women’s Development Army (WDA). | Quantitative | 422 | volunteer |
| 20 | Keno M., 2021 | Ethiopia | To determine the level of turnover intention and to identify factors associated with turnover intention among health extension workers | Mixed methods | 245 health extension workers for quantitative; 6 key informant for qualitative | paid |
| 21 | Li Li., 2014 | China | To examine levels of work stress and motivation and their contribution to job satisfaction among community health workers | Quantitative | 930 | paid |
| 22 | Maryse C., 2015 | Ethiopia | To understand how relationships between HEWs, the community and health sector were shaped, in order to inform policy on optimizing HEW performance in providing maternal health services. | Qualitative | 175(FGD, and interview participants). From these HEWs are 63 | paid |
| 23 | Medhin S., 2017 | Ethiopia | To explore the conceptualizations of wellbeing, stress and burnout among healthcare workers in primary healthcare settings in rural Ethiopia | Qualitative | 52(17 are CHWs) | paid |
| 24 | Mokholelana M., 2020 | South Africa | To describe the role of CHWs in community-based health care in Northern Cape, Identify the perceived barriers and enablers to CHWs role performance, Explore CHWs views regarding the support from the communities and the formal healthcare system in Northern Cape | Qualitative | 46 | paid |
| 25 | Olatunde A., 2021 | Nigeria | To determine whether non-clinical Community Health Workers (called Community-Oriented Resource Persons, CORPs) implementing iCCM could use simplified tools to treat uncomplicated SAM | Mixed methods | 60 | volunteer |
| 26 | Pascal G., 2017 | Swaziland | To determine what potential changes to their program CHWs and CHW program managers perceive as likely leading to improved performance of the CHW cadre | Qualitative | 54 | paid |
| 27 | Phillip W., 2016 | Uganda | To assess the performance of CHWs in managing malaria, pneumonia, and diarrhea | Mixed methods | 393 | volunteer |
| 28 | Rosalind M., 2016 | Kenya | To explore the drivers of policy change from the perspectives of policymakers and SCHMT members, as well as the perceptions of the current system from community to sub-county level | Qualitative | 40 In-depth interviews, and 10 focus group discussions. | volunteer |
| 29 | Ruth J., 2019 | Ethiopia | To apply a gender lens to Ethiopia’s Health Extension Program and the role of Health Extension Workers | Qualitative | 76 | paid |
| 30 | Saji S., 2012 | India | To examine the performance and motivation of community health workers and its determinants | Mixed methods | 386 | volunteer |
| 31 | Samuel C., 2019 | Ghana | To assess the level of performance and factors that affect the performance of health volunteers’ activities | Quantitative | 200 | volunteer |
| 32 | Sara J., 2011 | Iran | To explore the perceptions of CHWs regarding their contribution to rural health | Qualitative | 91 | paid |
| 33 | Sarah S., 2014 | Malawi | To understand the performed versus documented roles of the HSAs, to examine how tasks were prioritized, and to understand HSAs’ perspectives on their roles and responsibilities | Qualitative | 70 HSAs (CHWs) | paid |
| 34 | Shyam L., 2021 | Ethiopia | To estimate the job preferences of public health sector community health extension workers, care providers including nurses and midwives, and non-patient-facing administrative and managerial staff | Quantitative | 390 total (198 CHWs) | paid |
| 35 | Syed M., 2010 | Bangladesh | To investigate reasons for high rates of CHW attrition | Mixed methods | 69 | paid |
| 36 | Seutloali T., 2018 | Lesotho | To explore the lived experience of CHWs in conducting health promotion activities | Qualitative | 36 total(32 with CHWs; 4 with CHWs' supervisors) | volunteer |
| 37 | Wilson M., 2020 | South Africa | To explore intrinsic and extrinsic factors that CHWs face | Qualitative | 20 CHWs | paid |
| 38 | Yared A., 2018 | Ethiopia | To explore barriers and facilitators to timely postnatal visits | Qualitative | 20 CHWs (in 4 FGDs); 19 interviews with mothers and close relatives. | paid |
| 39 | Yueh-Mei G., 2013 | Taiwan | To examine the burden experienced by community health volunteers in Taiwan. | Quantitative | 435 | volunteer |
| 40 | Yusufu K., 2017 | Uganda | To describe the socio-demographic and workplace characteristics affecting CHVs performance in a public health program | Quantitative | 508 | volunteer |
| 41 | Kabula J., | Tanzania | To explore challenges the CHWs face in promoting maternal and neonatal health in two districts of Tanzania | Qualitative | 30 | volunteer |
| 42 | Ambimbolla O., 2021 | Bangladesh, India, Kenya, Malawi and Nigeria | To explore factors influencing motivation and satisfaction among CHWs in LMICs | Qualitative | 32 FGD, 116 key informant interviews | Volunteer (Nigeria Kenya, India); Paid (Bangladesh, Malawi) |
| 43 | John P., 2014 | Malawi | To evaluate the current jobs status of community health workers and their potential to implement integrated approaches. | Qualitative | 67 | paid |
